# Association of variable number of tandem repeats (VNTR) and T941G polymorphism of monoamine oxidase (MAO-A) gene with aggression in Pakistani subjects

**DOI:** 10.4314/ahs.v21i1.24

**Published:** 2021-03

**Authors:** Sumbal Sarwar, Shabana, Shahida Hasnain

**Affiliations:** Department of Microbiology and Molecular Genetics, University of the Punjab, Lahore, Pakistan, 54590

**Keywords:** Aggression, MAO-A gene, VNTRs, T941G, rs6323, Pakistan

## Abstract

**Background:**

Human behavioral traits are known to be significantly heritable. Certain individuals have a greater tendency of negative behavioral aspects including aggression. The quest to identify tunderlying genetic causes has led to identification of a number of genetic markers, one of them is the monoamine oxidase-A (MAO-A) gene.

**Objective:**

We aimed to genotype a variable number of tandem repeats (VNTRs) in the promoter region and a functional SNP within this gene (T941G, dbSNP ID: rs6323) in the recruited cohort of 482 subjects.

**Methods:**

After DNA isolation, genotyping was done by PCR-RFLP and the results were confirmed by sequencing.

**Results:**

For VNTRs, the results showed, highest frequency of 3.5 repeats in males and 4 repeats in females in the promoter region. The genotype frequencies for the SNP in cases were GG=16.3%, TG=20.6% and TT=63.1%, while in controls, the frequencies were GG=12.7%, TG=6.3%, and TT=81.0%. The allele frequencies were significantly different between cases and controls (p=0.015; OR=1.51; CI=1.085–2.102).

**Conclusion:**

The selected VNTR and SNP appeared to be significantly associated with aggression. These VNTRs and SNP have not been studied previously in the Pakistani population, hence they represent a unique ethnic group. These results, however, would have to be replicated in larger cohorts.

## Introduction

Human behavior is a collection of various emotions, instincts and characteristics that may have positive or negative impact not only on the bearer but also on other individuals of the society 1,2. Aggression is one of the most important traits of human behavior. It is a series of deliberate actions that leads to anguish, destruction or injury to other organisms. It promotes violence in society, imposing maximum harmful effects to the targeted person or victim 3. Behavioral genetics is based on the idea that every individual exhibits distinct interaction of genes in the environment 4. Environmental conditions influence upregulation or downregulation of genes. In the context of aggression, the most extensively studied gene for the gene-environment interactions is monoamine oxidase (MAO), more exclusively mentioned as monoamine oxidase-A, MAO-A (sometimes called “warrior gene”, “criminal gene” and “aggressive gene”; however such nomenclature remains disputed)^5^–^7^.

Monoamine oxidases are the mitochondrial enzymes that catalyze the oxidative deamination of several biogenic amines in the brain and certain peripheral tissues of the body by the production of hydrogen peroxide^8^. On the basis of substrate selection and inhibitor sensitivity, two forms of monoamines are classified as monoamine oxidase A and B (MAO-A and MAO-B). MAO-A is the gene that encodes for monoamine oxidase A and MAO-B is a gene which encodes for monoamine oxidase B. Both genes are paralogous and have been derived from the same ancestral gene 9,10. The chromosomal location of MAO-A and MAO-B is Xp11.3 and Xp11.3, respectively 11,12. The two enzymes vary in their substrate selection and action of inhibitors. MAO-A has a higher affinity for the substrate norepinephrine (NE), dopamine (DA), and serotonin. Inhibitors of MAO-A include clorgyline. MAO-B has a preference for the degradation of benzylamine and phenylethyleamine (PEA) and its inhibitor is deprenyl^13^. People with the low activity form (MAOA-L) have less amount of MAO-A enzyme. On the other hand, people with high activity form (MAOA-H) produce high amounts of MAO-A enzyme 14. Studies have demonstrated that low activity of enzyme MAO-A elevates the level of dopamine leading to aggression^5^. There is a list of physiological, psychological and behavioral disorders that are associated with monoamine oxidase A gene (supplementary table 1).

Among numerous variants of the MAO gene, the notorious one is the variable number of tandem repeats (VNTR), located in the promoter region of the gene. It is the most extensively studied polymorphic marker of this gene and has been reported to be associated with the aggressive behavior 15. These tandem repeats are situated 1.2kb upstream the coding region and generally contain four different alleles i.e. 3, 3.5, 4 and 5 repeats of 30bp (Supplementary Fig 1) 16. Rarely humans have an allele with 2 tandem repeats 16. These repeats affect the expression of the gene and also the activity of the enzyme. In the general population, 3 and 3.5 repeats are responsible for the low activity of the enzyme, whereas, 5 and 4 repeats are responsible for the high activity of the enzyme^15^, ^17^. None the less, there are many contradictions in the correlation between the number of repeats and activity of the enzyme 18.

A number of polymorphisms have been reported in the monoamine oxidase A gene (MAO-A). According to Ensembl, the total number of variants in the exons is 177 that includes stop gained, missense, frame shift, splice region, start lost and coding sequence variation. Thousands of variations are present in the intronic and regulatory regions of this gene. Previous studies identified a functional polymorphism, T941G, db SNP ID: rs6323, CGT297CCG) in exon 8 of MAO-A gene, whereby T is the ancestor allele while G is the polymorphic allele 19. The presence of G at position 941 creates restriction site for the enzyme Fnu- IV. Although this polymorphism is present at 3rd base of codon resulting in no change of amino acid in the protein, the substitution results in altered enzyme activity 17.

Previous studies have reported the association of this polymorphism with aggressive behavior, violence and antisocial alcoholism 20. The association of the number of tandem repeats and aggressive behavior is poorly understood in the Pakistani population and ethnic variation in allelic frequency is a major confounding factor in genetic studies that leads to false positive results. Therefore, we aimed carried out the study to determine the association of VNTR and association patterns of T941G of MAO-A gee with aggression in the Pakistani population.

## Materials and methods

### Study participants

Samples were collected from the general population of Pakistan. We randomly collected 330 unrelated samples from selected areas of the Punjab, Pakistan and 241 controls. The study protocol for human subjects was approved by the ethics committee (Ethical Committee, School of Biological Sciences SBS) of University of Punjab (SBS390/17). After obtaining informed written consent, all subjects were interviewed to assess their self-reported aggression by using a comprehensive questionnaire based on the State Trait Anger Expression Inventory (STAXI)^21^. The questionnaire consisted of 44 items that were distributed in 5 subsections, namely 1) state anger (10 items) 2) trait anger (10 items), which was further subdivided into anger temperament and anger reaction. Trait anger is subdivided into 10 items and angry temp, angry reaction are among subjects reporting aggression 3) anger-in (8 items), 4) anger-out (8 items), 5) angry control (8 items). All items are rated on a scale (1–4 score) from modest to strong. Confirmatory factor analysis and exploratory factor analyses were done that excluded 89 individuals from cases (330) so 241 cases remained. To campare with the cases, we collected 241 controls. A complete performa of subject's information, questionnaire and a consent form was taken from every individual.

### Determination of blood glucose levels

Blood glucose was measured using digital glucometer (Acu-check®). The participant's finger was sterilized with the help of spirit, skin puncture was done by a sterile lancet, a drop of blood was taken on the glucometer strip and the reading was noted within 1min.

### Genotyping

Five mililiters of blood was drawn from the participants. Half the blood was poured into disodium EDTA (purple caped) vials (to avoid blood clotting) for isolation of DNA. Genomic DNA was isolated from blood leukocytes using the Wizard® Genomic DNA purification kit (Promega, USA). All DNA samples were quantified and standardized (10ng/µl) prior to genotyping using Epoch Biotek microplate reader.

Standard PCR for VNTR and T941G was done in advanced primus 96 (PeqLab) thermal cycler to amplify the isolated genomic DNA with Thermo Scientific Mastermix (Cat#K0171). For VNTR, we used primers as described previously^22^. The primers' sequences were: forward 5′- ACAGCCTGACCGTGGAGAAG -3′ and reverse 5′- GAACGGACGCTCCATTCGGA -3′. The PCR product size was 291–381bp. PCR reaction conditions consisted of initial denaturation at 95°C for 2 min, 35 cycles of denaturation at 94°C for 1min, annealing at 55.5 for 1min, extension at 72 for 2min and a final extension at 72 for 5min. For T941G we also used published primers^23^. The primer sequence was forward 5′- GACCTTGACTGCCAAGAT -3′ and reverse 5′- CTTCTTCTTCCAGAAGGCC -3′. The PCR product size was 130bp. PCR reaction conditions consisted of initial denaturation at 95°C for 3 min, 35 cycles of denaturation at 94°C for 1min, annealing at 64°C for 1min, extension at 72 for 2min and a final extension at 72 for 5min. Restriction fragment length polymorphism (RFLP) was done for all samples to determine the presence of SNP. The PCR product 130bp was digested with the help of Fnu 4HI restriction enzyme. A vector, pBR322 was used as a positive control for the digestion reactions. The time for incubation was 8–16 hour. Finally the PCR products of VNTR, T941G and digested products were seen by running 2% agarose gel electrophoresis and observing the gel under Gel Doc system/U.V light. The results of RFLP analysis were confirmed by direct sequencing. Both strands of selected PCR product were sequenced with a BigDye® Terminator v3.1 from Korea. Sequencing has been done to confirm the results of RFLP analysis.

### Statistical analysis

Microsoft Excel and Statistical Package for Social Sciences (SPSS, IBM statistics version 22) software were used for statistical analysis. Anthropometric, biochemical and genotype data were analyzed for means and standard deviations. The study population (recruited cases and controls) was tested for Hardy Weinberg Equilibrium (HWE). A p-value of <0.05 was used as a significance cutoff for all analyses. All procedures adopted either for isolation of DNA or the downstream process were according to international biosafety standards. Personal protective and laboratory safety measures were strictly observed and all the materials that were hazardous or contaminated were properly disposed off.

## Results

### Characteristics of study subjects

This study included 482 individuals. Confirmatory factor analysis and exploratory factor analyses were done that excluded 89 individuals from cases (330) so 241 cases remained. To campare the cases with controls, we collected 241 controls. So the total number was 482, from different areas of the Punjab, Pakistan. All study participants were above 10 years. All infectious samples were excluded. In the samples, 60% (145/241) were males and 40% (96/241) were females; and in the controls there were 51.08% (123/241) males and 48.91% (118/241) females. Among the samples, mean age was 39.58±13.0 while in the controls mean age was 33.54±12.1 years. Among the samples, mean weight was 63.03±12.53 while in controls mean weight was 61.00±14.7 kg. Mean height in the samples was 5.5±0.36 while in controls mean height was 5.5±0.29 meters. Mean value of BMI in the samples was 22.1±5.33, on the other hand, in controls mean BMI was 21.6±6.37 (table 1).

**Table 1:** Characteristics of study samples

Characteristics	Controls	Samples
**Male**	51.08%	60%
**Female**	48.91%	40%
**Age (Years)**	33.54±12.1	39.58±13.0
**Weight (kg)**	61.00±14.7	63.03±12.53
**Height (m)**	5.5±0.29	5.5±0.36
**BMI (kg/m^2^)**	21.6±6.37	22.1±5.33
**Systolic (mm/Hg)**	118.9±13.1	116.93±12.0
**Diastolic (mm/Hg)**	83.2±8.1	76.27±8.62
**Glucose level (mg/dl)**	138.02±34.8	135.5±32.0

### VNTR of MAO-A gene

**Figure 1:**
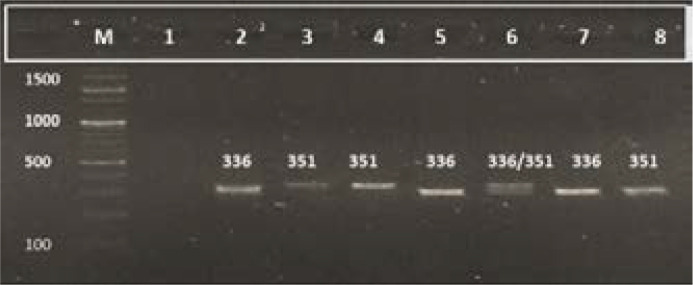
Amplification of genomic DNA for VNTR on 2% agarose gel, The size of amplified product is from 336 to 351bp and all wells in the picture contain different sizes of bands. From 2 to 8 wells containing amplified product, well 6 contains heterozygous repeats. In M there is a marker, for the determination of size of amplified products. The lowest band size of the marker is of 100bp and the largest band is of 1500bp. Marker/Ladder was used to identify the approximate size of a DNA bands run on a gel during electrophoresis.

Followed by the amplification of genomic DNA, gel electrophoresis was done. Variable number of tandem repeats (VNTR) were noted in the cases as well as in the controls (Figure 1). According to the results, the group of male samples had the highest frequency of 3.5 numbers of repeat; and females had the highest frequency of 4 repeats. In controls, males had the highest frequency of 3 numbers of repeats; and females had the highest frequency of 5 repeats. The distribution of different VNTR alleles among the subjects is shown in Table 2.

**Table 2:** VNTR repeats in the study population

VNTR Allele	Number	Percentage %
**2**	4	1.4
**3**	66	23.4
**4**	67	23.8
**5**	38	13.5
**3.5**	72	25.5
**3/3.5**	1	0.4
**4/3.5**	17	6.0
**5/3.5**	6	2.1
**4/3**	10	3.5
**5/3**	1	0.4

### Allele and genotype frequency of T941G variant

**Figure 2:**
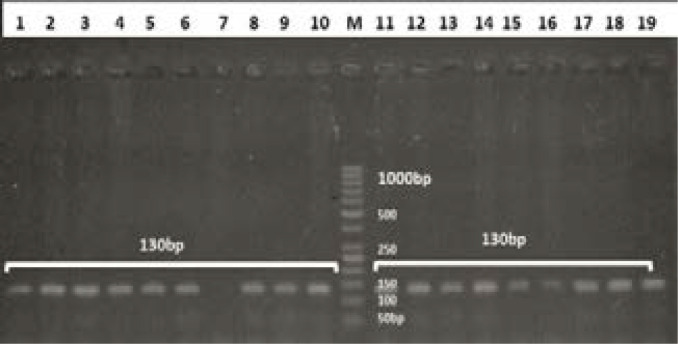
Amplification of genomic DNA for polymorphism T941G (rs6323) on 2% agarose gel, the size of amplified product is 130bp and all wells in the picture contain same size of band. From 1 to 10 and 11 to 19 wells containing amplified product. No product in well 7. In M there is a marker, for the determination of size of amplified products. The lowest band size of the marker is of 50bp and largest band is of 1000bp.

**Figure 3:**
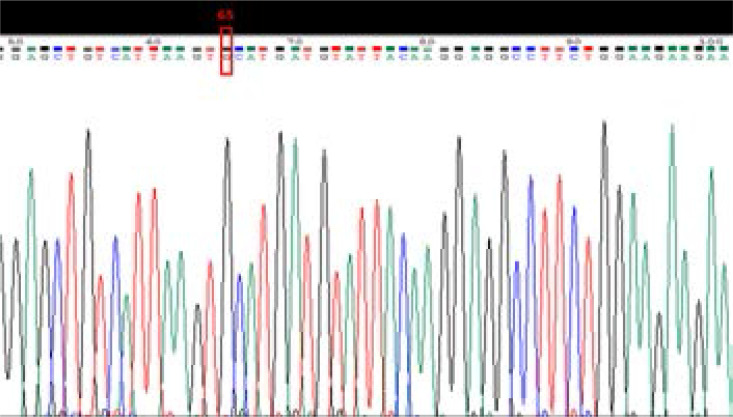
Sequence analysis of an amplified product of polymorphism T941G (rs6323), after sequencing, this sequence contains polymorphic allele **G** at position 65

**Figure 4:**
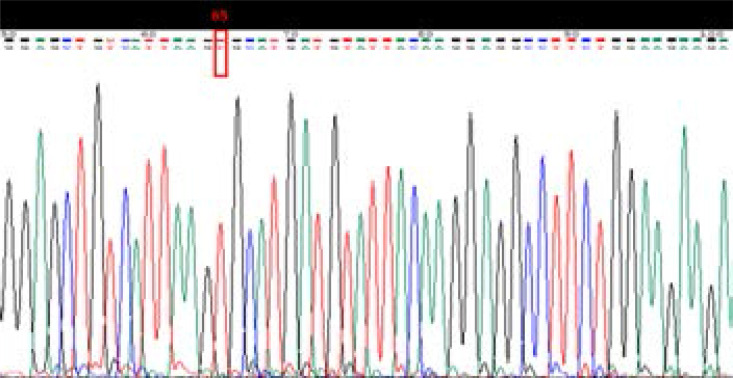
Sequence analysis of an amplified product of polymorphism T941G (rs6323), after sequencing, this sequence contains ancestor allele T at position 65

For MAO-A gene polymorphism T941G (rs6323) genotyping, PCR-RFLP genotyping method was used (Figure 2). The results were confirmed by sequencing of random samples (Figure 3 and 4). Significant differences were observed in allele frequencies between the cases and the controls (p=0.015, OR=1.51 CI=1.09–2.10) (Table 3). The allele frequencies in the controls were G=73.4% and T=84.2%, while in the cases were G=26.6 and T=15.8%. The genotype frequencies in the cases were GG=16.3%, TG=20.6% and TT=63.1%, while in the controls they were GG=12.7%, TG=6.3%, and TT=81.0% (Table 4). The polymorphism was significantly associated with aggressive behavior as tested by logistic regression (p=0.015).

**Table 3:** Allele frequency in study population

Allele	Controls	Samples	*p*- value	OR (Odds ratio)	95 % CI	
					Lower	Upper
**T**	84.2%	15.8%	0.015	1.510	1.085	2.102
**G**	73.4%	26.6%				

**Table 4:** Genotype frequency in study population

Genotype	Aggressiveness		Total
	Controls	Samples	
**TT**	148	123	**271**
**TG**	42	62	**104**
**GG**	51	56	**107**
**Total**	241	241	**482**

## Discussion

Pakistan is a low income country, currently facing many issues 24,25. According to a survey, 17.2% of Pakistani population is living below the poverty line^26^–^28^. These circumstances along with other crisises make the environment of this country favorable in provoking aggressive behavior in the general population. In humans, the way we look and behave is determined by the complex interaction of hundreds of complex traits, each of which is in turn governed by a combination of inherited factors, both genetic variants and environmental stimuli.

VNTR repeats in the promoter region of the MAOA gene have been frequently studied in different populations^6^,^16^, ^29^–^33^, as well as in different ethnic groups worldwide but have not been studied in the Pakistani population yet. Hence, the current study is the first report to unravel the association of VNTR of MAO-A gene with aggression in this unique ethnic group. Our results showed variation in the number of variable tandem repeats in the study group. A previous study had shown that alleles with 4 and 3.5 copies of the repeat sequence are transcribed more efficiently than those with 5 or 3 copies of the repeat sequence 17. In our study group of male samples had highest frequency of 3.5 number of repeat and of female samples had the highest frequency of 4 repeats. In the control male samples, there was a highest frequency of 3 number of repeat and in control female samples the highest frequency was of 5 repeats. According to the current study VNTR of MAO-A gene is associated with aggression in the Pakistani population. Some of the previous studies state that males tend to be more aggressive naturally^34^,^35^.

A number of variations are present in intronic and regulatory region of the MAO-A gene. Studies have revealed that T941G is a functional polymorphism (exon 8) of monoamine oxidase A gene especially among Asian population^23^, ^36^–^39^. An initial analysis of aggressive samples revealed a greater number of males (60%) than females (40%). It does not mean that aggression is more common in males than females, it just corresponds to the more availability of male samples and a greater degre of consent to give blood samples by males than females. It was more difficult to convince females to give samples due to illiteracy; however, almost all subjects who voluntarily gave the samples were willing to follow up and know the outcome of the study. Lastly, in the light of analysis done using collected samples, 60% of males and 40% females were found to be aggresive. For MAO-A gene polymorphism T941G (rs6323) PCRRFLP genotyping method was used and significant differences were observed for allele (p=0.015, OR=1.510 CI=1.085–2.102). The polymorphism was explicitely associated with aggressive behavior as tested by logistic regression (p=0.015).

The study of polymorphism T941G (rs6323) was also done by the 1000 human genome project (Supplementary Figure 2: http://www.internationalgenome.org/1000-genomes-browsers). According to the 1000 human genome project, the allele frequency in African population was G=0.138 and T=0.862 and the number of samples analysed was 661. The allele frequency in American population was G=0.294 and T=0.706 and the number of samples analysed was 357. The allele frequency in East Asian population was G=0.575 and T=0.425 with 504 samples being analysed. The allele frequency in European population was G=0.287 and T=0.713 with 503 samples being analysed. The allele frequency in South Asian population was G=0.648 and T=0.352 found by analyzing 489 samples. The allele frequency in PJL (Punjabi in Lahore), Pakistan population was G=0.611 and T=0.389 reported by analyzing 96 samples.

In comparison to PJL, our study resulted in genotype frequency in cases G=0.226 and T=0.734 and in controls G=0.158 and T=0.842. In PJL the sample size was 96 and we had 141 cases and 141 controls. So the sample size of our study was comparatively higher (282) than PJl study of 1000 human genome project. The samples included in PJL were all controls and it was not a case control study for the polymorphism. In 1000 Human genome project, the the samples from different ethnicities were sequenced. According to the statistical analyses in the current study, the results of genotypic frequency were in accordance with the African, American, and European population and in contrast with the East Asia, South Asian and PJL population (1000 human genome project http://www.internationalgenome.org/1000-genomes-browsers) [P6][P7]The contradictory findings may be attributed to different sample sizes, possible population stratification, and ethnic differences.

Our results revealed that the polymorphism is significantly associated with aggression in local population (p=0.015). The minor allele or risk allele, G appeared to increase the risk of aggression in the studied population, while the major allele T behaved as protective allele as it had a higher frequency in control samples in comparison to aggressive samples. The polymorphism T941G was studied widely from 2006 to 2015 in different population in association with autism spectrum disorder, schizophrenia, suicidal attempts, antidepressant response, behavioral problems, major depressive disorder and borderline personality disorder (supplementary table 2).

## Conclusion

Although it is very difficult to precisely unravel the genetic and environmental factors of various aspects of human behavior, the investigation of the ethnicity specific genetic markers can provide useful insights about the role of genetic factors underlying these behavioral traits. The SNP (T941G -rs6323) and VNTR of MAO-A gene being studied showed a vivid association with aggression in the local population. The study sample size was relatively small, albeit the positive results point toward the possible role of modest effect size common variants in aggression as well as may explain the inter-individual variation in the extent and type of aggressive behavior. The current results encourage a further investigation of this SNP (T941G -rs6323), VNTR of MAO-A gene and other genetic markers in a larger cohort of Pakistani population.
